# Dyslexia: Still Not a Neurodevelopmental Disorder

**DOI:** 10.3390/brainsci9010009

**Published:** 2019-01-10

**Authors:** Athanassios Protopapas, Rauno Parrila

**Affiliations:** 1 Department of Special Needs Education, University of Oslo, Oslo 0318, Norway; 2 Department of Educational Studies, Macquarie University, Sydney 2109, Australia; rauno.parrila@mq.edu.au

**Keywords:** dyslexia, reading difficulty, brain, neurodevelopmental disorder, neuroimaging

## Abstract

We recently pointed out that there is no evidence to support the commonly held view that there is something wrong with the brains of children who have great difficulty learning to read. In response, it was argued that dyslexia should be considered to be a neurodevelopmental disorder because of its potential to adversely affect quality of life, and because there are differences between the brains of people with different levels of reading skill. We agree with these two points, but they are irrelevant to the issue in question, because neither establishes the critical notion of disrupted neurodevelopment; that is, a brain fault. Differences between groups do not imply that any individuals are abnormal, and calling a brain improperly developed on the basis of cultural issues has absurd implications. Even calling brains atypical is unfounded because reference to typicality hinges on knowledge of the relevant distributions, which is currently lacking. Moreover, there is at present no obvious role for neurology- or neuroscience-based input for the critical issue of the assessment and remediation of the reading difficulties themselves. We reiterate our conclusion that there is, at the moment, no credible foundation to support the claim that dyslexia is a neurodevelopmental disorder.

## 1. Introduction

In our recent article [1] (henceforth PR18), we pointed out that there is no evidence to support the commonly held view of dyslexia as a neurodevelopmental disorder. That is, there is no evidence to suggest that there is anything wrong with the brains of children who have great difficulty learning to read despite normal sensory function and educational opportunity. Fraga González, Karipidis, and Tijms [2] (henceforth FKT18) have taken issue with our view, coming forth with a response purporting to establish the notion of dyslexia as a neurodevelopmental disorder. However, it seems to us that they simply perpetuate the sloppy thinking and misinterpretation of data that is rampant in the field. Although FKT18 make no novel points that would require us to revise or augment our arguments, we believe it is important to respond in order to clarify matters, as it seems that our original contribution has been insufficiently effective in dispelling certain fundamental misconceptions. We hope that, in the present brief rejoinder, we can more successfully explain what the main issues and problems are.

## 2. Points of Convergence

Before focusing on our points of disagreement, let us point out some very substantial points on which we believe we are in agreement with FKT18. First, there is indeed harm: there is no question that poor reading is a serious problem and that it requires special attention in the form of assessment and remediation as early as possible. This is a non-negotiable primary concern for us, as it also seems to be for FKT18, and in this respect, it can be the basis for any further discussion. The downstream all-too-tangible consequences and secondary costs associated with dyslexia are well known (e.g., [3,4,5,6]) and it is in the interest of minimizing them that we participate in these discussions. We believe we were very clear about this in PR18 and, in this sense, FKT18’s argument that dyslexia differs from a chess disorder (one of our examples) in that it has serious consequences cannot be directed against us but must concern other agents. Similarly, there is no disagreement stemming directly from the continuous nature of reading skill. Dyslexia can be a “continuous disorder” [7] as is the case with obesity and high blood pressure. The problem is, as we stated in PR18, that “there is no evidence demonstrating that one end of a purported neural continuum, presumably reflecting the behavioral continuum, represents outcomes of developmental failure” (p. 13). Moreover, we suspect that some people may find the neurodevelopmental disorder notion tempting precisely because they fail to appreciate the continuity, having a more black-and-white notion in mind.

Second, dyslexia exists and it is biological, in the sense that there is a persistent and unexpected difficulty in developing age- and experience-appropriate word reading skills (see [8] for clarifications and elaboration) and this difficulty is accounted for by an individual’s brain structure and function. Or, as we put it in PR18, “it is brain structure and function that determines reading skill, including the highest and lowest levels of skill and all those in between” (p. 13; see PR18 for clarifications and elaboration). In particular, brain structure and function must be associated with current level of reading skill (in readers) as well as with the propensity to acquire the skill (in pre-readers). This is an inescapable conclusion given our understanding of the brain as the physical substrate of behavior, whereby brain function is taken as constitutive of the perceptual and cognitive processes amounting to the act of reading, as much as to the process of learning to read. Indeed, we consider the findings of associations between the brain and behavioral differences to be trivial as such. In this sense, FKT18’s reference to a multitude of studies demonstrating associations between neuroimaging (or other brain-based or genetic) measures and level of reading skill can hardly be directed against our argument.

Given that most of FKT18 concerns precisely these two points, one may be left wondering what the fuss is all about, and how can we have such different worldviews if we agree on the two most fundamental issues. We share this puzzlement. The problem seems to be that FKT18 appear to draw different conclusions which, we believe, are wholly unsupported by the available evidence and generally unfounded. Our main point in PR18 was that there is no evidence to support the idea that dyslexia is a neurodevelopmental disorder. The term “neurodevelopmental disorder” refers to disrupted neurodevelopment; that is, a developmental failure of some sort. In other words, something in the brain has not developed properly and, as a result, there is something wrong in it. The claim that dyslexia is a neurodevelopmental disorder can only be supported by findings demonstrating faults in brains—that is, specific things in specific brains which have gone wrong in specific ways—and, by definition, nothing else qualifies. However, nothing in FKT18 addresses this crucial issue.

## 3. On the (Mis)Interpretation of Neuroimaging Findings 

In particular, in PR18, we made two main points with respect to neuroimaging, both of which are ignored by FKT18 in their response. Specifically, we pointed out that brain differences must necessarily underlie behavioral differences. The brains of a group of poor readers must be different from the brains of a group of good readers, the difference in brains underlying—and corresponding to—the difference in reading skill. Likewise, the brains of a group of good singers must be different from the brains of a group of people who cannot sing, the brains of a group of good chess players must be different from the brains of a group of people who play chess badly or not at all. This is trivial, and, by extension, it is trivial to demonstrate differences between groups of differentially skilled people. However, most neuroimaging findings to date have demonstrated precisely such differences and have been incorrectly and misleadingly claimed to be demonstrating anomalies. 

As we noted, “different” is not the same as “wrong”. Demonstrating differences does not imply that either group of brains is improperly developed, or improperly wired, or improperly functioning: a difference is just a difference. If one wishes to demonstrate abnormality, then a completely different type of work is necessary to establish that well-specified brain properties are outside of some independently established, brain-based and brain-specific criteria. Moreover, this has to be determined before the onset of skill acquisition, because if it is observed in already-skilled persons, who must have practiced for a long time in trying to acquire the skill, then whatever is found may well be an outcome, rather than a cause, of the skill level. None of the studies cited by FKT18 meet these criteria. Indeed, it is currently impossible to meet these criteria because it remains entirely unknown what is required for the neurodevelopment for reading skill to develop unimpeded. Instead, FKT18 simply list another set of studies demonstrating theoretically trivial correlations between brains and behaviors, with no bearing on the critical issue of developmental failure.

Our second main point had to do with the notion of typicality, which is ignored by FKT, as it has been blissfully ignored by the entire field. Simply put, a difference in the average value between two groups has no bearing on any individual member of either group, because significant and reliable differences can be obtained in the presence of a great overlap in the actual values between the two groups. In other words, “typical” is not the same “average”. Consider, for example, height: that men are reliably taller than women, on average, is a robust empirical fact expressing a reliable group difference. This fact would not cause us to consider that women, as a group, are abnormally short, it would not cause us to expect every woman to be shorter than every man, and it would not cause us to expect every woman to have the average women’s height. We are well acquainted with the fact that there are many women who are as tall as the average man, as well as women who are taller than that. This blatantly obvious set of facts somehow eludes us when we are thinking of brain activations rather than heights, so that some of us seem perfectly happy to conclude that dyslexics’ brains are abnormal or atypical on the basis of an empirical finding of some average difference. 

Beyond this trivial fallacy, the problem of interpreting differences in functional neuroimaging is actually much worse because of an additional discrepancy affecting what is reported as group-average patterns of activation, which is little-appreciated outside the field. Specifically, the usual methods used to analyze the signals coming out of the MRI scanners concern the magnitude of the signal at a series of individual locations in the brain (actually, at a set of “elementary volumes” a few millimeters across). Yet our talk of “activation patterns” refers to spatial interpretations; that is, to how the locations of signal difference are distributed over the brain’s surface or volume. We look at pictures with the statistical results displayed as colored blobs here and there, and we think of these blobs as the pattern of activation. However, the presence of the blobs in one or another location has arisen because the individual locations have crossed some threshold in a group analysis of signal magnitude. 

This means two things: first, even a tiny difference in signal quantity can substantially shift what we perceive as a “pattern” if it causes blobs to cross the threshold. Blobs narrowly missing the threshold remain unreported and thus invisible to meta-analyses. Second, and most importantly, what comes out as an average pattern of blobs concerning a group, or difference between groups, has little bearing on the actual patterns of activation in individual brains, because the average only reflects the consistent locations. In other words, locations of activation that may be strong and robust for only one or a few persons are bound to disappear in the average picture if they are not exhibited by a majority of participants. This is because activations are averaged numerically, not spatially, under the assumption that patterns are largely shared across people—an assumption that is rarely evaluated and most likely false for any complex operation such as reading (see PR18 for references and an example). Thus, our existing analyses, and their results, tell us absolutely nothing about what a typical pattern of activation is. We literally do not know if individual typically developing readers exhibit similar patterns of activation that can be ipso facto termed typical, or if some (or even most) of them exhibit patterns of activation that are very different from the group average. As a result, it is impossible to tell whether any good or poor readers have patterns of activation that are atypical, because the distribution of activation patterns in the population is unknown since nobody has studied it. Therefore, every proclamation that an atypical activation pattern has been demonstrated is necessarily incorrect and misleading. Yet FKT18 follow the standard practice of misinterpretation, evidently unassailed by these considerations, by referring to atypical development of neural networks and to brain abnormalities.

## 4. Further Implications and Objections

Instead of presenting relevant evidence to support the assertion that dyslexia is caused by disrupted neurodevelopment, FKT18 claim that viewing dyslexia as a neurodevelopmental disorder provides “an instrumental framework to plan informed interventions” (p. 2). However, there is no hint at what such interventions might be, and why alluding to fictional brain faults would help plan them. What, exactly, would the role of the neurologist be in the planning of reading interventions? 

This claim is absurd on both ends: first of all, the need for interventions arises from the poor reading itself, regardless of whether a brain fault exists or not. If it turned out in the future that a brain fault does exist after all that is reliably associated with dyslexia, would a reading intervention be provided to someone with no reading difficulty on the basis of the brain fault alone? More importantly, would a reading intervention be withheld from someone with reading difficulty if they failed to exhibit the fault in question? If the latter question is generally answered positively, then it is clear which approach—ours or FKT18’s—deprives poor readers of necessary support; if not, then the brain fault is patently irrelevant. 

Second, once the need for intervention is established, the success of the intervention can only be judged behaviorally. If there is an improvement in reading performance, the intervention is successful, whereas a lack of improvement renders the intervention unsuccessful, regardless of any concomitant neural changes of the predicted sort (or lack thereof). Thus, the claimed “instrumental framework” is not just conspicuously absent, it is at present no more than a pipe dream. Granted, it is conceivable that, at some point in the distant future, the nature of a brain fault may be established which might help guide the choice between different interventions, thereby increasing the likelihood of success. At the moment, however, all such choices can only be based on behavioral rather than neural information.

Finally, let us point out an absurd corollary of the idea that difficulty acquiring a skill should be called a “neurodevelopmental disorder” to the extent it negatively impacts quality of life, as FKT18 suggest. This amounts to deciding what a neurodevelopmental disorder is (i.e., whether a brain has developed properly or not) on the basis of the contemporary cultural ambience. A brain that is not well suited to learning to read is abnormally developed in our contemporary society, but would have been perfectly normal a few thousand—or even dozen—years ago, or even today in an illiterate society (or perhaps even in another linguistic context [9]). Alternatively, if we stick to the notion that the same brain cannot be both normal and developmentally disrupted at the same time, then every brain that is not suited for learning to read must have always been abnormal. It is just that people in preliterate societies lacked the cultural opportunity to fail to learn to read and thereby to find out that their neurodevelopment was in fact disrupted. 

The problem with this view is that one never knows what might come into vogue after a millennium or two, such that difficulty acquiring it might severely impact one’s quality of life. This inevitably leads to the conclusion that we cannot ever be sure that any brain is in fact properly developed, because it may be unsuited for learning something that will become crucial in the distant future. That is, FKT18’s reasoning leaves everyone forever hanging in limbo between normal and neurodevelopmentally disordered before the vast realm of future cultural possibilities. We hope that the absurdity of this thought experiment is sobering with respect to the criteria that are relevant for deciding what counts as a neurodevelopmental disorder.

## 5. Conclusions

In summary, FKT18 fail to provide any evidence to support the notion that the persistent and unexpected difficulty in developing the age- and experience-appropriate word reading skills that is called “dyslexia” is due to frank neurodevelopmental failure. Instead, they claim that (a) dyslexia should be treated because of the potential to adversely affect quality of life, and that (b) there are differences between the brains of people with different levels of reading skill. However, these claims, with which we are in agreement, have no bearing on the issue in question. Thus, we maintain that there is, at the moment, no credible foundation to support the claim that dyslexia is a neurodevelopmental disorder. We suggest that, instead of searching for nonexistent brain faults, our efforts may best be focused on detecting the risk for reading failure as early and as reliably as possible, and on addressing the needs of persons with difficulty learning to read as early and efficiently as possible and as much as necessary.
